# Senescent peritoneal mesothelium creates a niche for ovarian cancer metastases

**DOI:** 10.1038/cddis.2016.417

**Published:** 2016-12-29

**Authors:** Justyna Mikuła-Pietrasik, Paweł Uruski, Patrycja Sosińska, Konstantin Maksin, Hanna Piotrowska-Kempisty, Małgorzata Kucińska, Marek Murias, Sebastian Szubert, Aldona Woźniak, Dariusz Szpurek, Stefan Sajdak, Katarzyna Piwocka, Andrzej Tykarski, Krzysztof Książek

**Affiliations:** 1 Department of Hypertensiology, Angiology and Internal Medicine, Poznań University of Medical Sciences, Długa 1/2 Str., Poznań 61-848, Poland; 2 Department of Pathophysiology; Poznań University of Medical Sciences, Rokietnicka 8 Str., Poznań 60-806, Poland; 3 Department of Clinical Pathology, Poznań University of Medical Sciences, Przybyszewskiego 49 Str., Poznań 60-355, Poland; 4 Department of Toxicology; Poznań University of Medical Sciences, Dojazd 30 Str., Poznań 60-631, Poland; 5 Division of Gynecological Surgery, Poznań University of Medical Sciences, Polna 33 Str, Poznań 60-535, Poland; 6 Laboratory of Cytometry, Nencki Institute of Experimental Biology, Polish Academy of Sciences, Pasteura 3, Warsaw 02-093, Poland

## Abstract

Although both incidence and aggressiveness of ovarian malignancy rise with age, the exact reason for this tendency, in particular the contribution of senescent cells, remains elusive. In this project we found that the patient's age determines the frequency of intraperitoneal metastases of ovarian cancer. Moreover, we documented that senescent human peritoneal mesothelial cells (HPMCs) stimulate proliferation, migration and invasion of ovarian cancer cells *in vitro*, and that this effect is related to both the activity of soluble agents released to the environment by these cells and direct cell-cell contact. The panel of mediators of the pro-cancerous activity of senescent HPMCs appeared to be cancer cell line-specific. The growth of tumors in a mouse peritoneal cavity was intensified when the cancer cells were co-injected together with senescent HPMCs. This effect was reversible when the senescence of HPMCs was slowed down by the neutralization of p38 MAPK. The analysis of lesions excised from the peritoneum of patients with ovarian cancer showed the abundance of senescent HPMCs in close proximity to the cancerous tissue. Collectively, our findings indicate that senescent HPMCs which accumulate in the peritoneum *in vivo* may create a metastatic niche facilitating intraperitoneal expansion of ovarian malignancy.

Incidence of ovarian cancer rises exponentially with age, irrespective of its histological type.^1^ Besides, aging determines the malignant potential of tumors and affects the 5-year survival of patients.^2, 3^ From the mechanistic point of view, the age-dependency of ovarian neoplasms is primarily linked with menopause-related overproduction of gonadotropins and the natural decline of gonadal steroids. Interestingly, low level of estrogens combined with high production of pituitary gonadotropins are particularly specific picture for early period after menopause and consistent with time when the incidence of ovarian cancer reaches the highest level. Another causative links between aging of endocrine system and ovarian cancer include the time-dependent accumulation of preneoplastic lesions within the ovary, plausibly combined with the depletion of ovarian follicles.^4, 5, 6^

The most life-threatening and unique feature of ovarian cancer is its predilection for the peritoneal cavity.^7^ Peritoneal tumors have been found to be developed in as much as 70% of patients in stage III or IV of the disease.^8^ It is believed that the intraperitoneal spread of the disease is governed by interactions between cancer cells and human peritoneal mesothelial cells (HPMCs).^9, 10^ Interestingly, pro-cancerogenic activity of HPMCs increases when the cells become senescent.^11, 12^

It is worth noting that the contribution of senescent HPMCs to the pathogenesis of ovarian cancer has never been studied in a comprehensive manner. This study was designed to verify our original theory that increased aggressiveness of ovarian cancer in elderly patients may be associated with deleterious paracrine activity of senescent HPMCs.

## Results

### Patient's age determines the intraperitoneal dissemination of ovarian cancer

The clinical histories of 111 women suffering from ovarian cancer were analyzed with respect to the effect of a given patient's age on the presence of peritoneal tumors. Two separate analyses were performed in this regard. In the first analysis the patients were arbitrarily grouped according to their age (⩽39 years; 40–59 years; ⩾60 years), while in the second analysis they were grouped according to their menopausal status (⩽51 years *versus* >51 years), assuming that the median age of natural menopause in Europe is between 50.1 and 52.8 years.^13^ In both cases the age of the patients was confronted with the stage of their disease according to FIGO grading, in which patients in stage I-II have no peritoneal spread, while those in stage III-IV are positive for peritoneal tumors.^14^

The results indicate that the percentage of patients having peritoneal tumors in the oldest group is almost two-fold higher as compared to that of the youngest patients. And, by contrast, the percentage of patients lacking peritoneal metastases remarkably declines in the oldest age group. The results obtained for the menopause-based criterion were analogical (Table 1).

### Senescent HPMCs promote the progression of ovarian cancer cells *in vitro*

**Figure 1:**
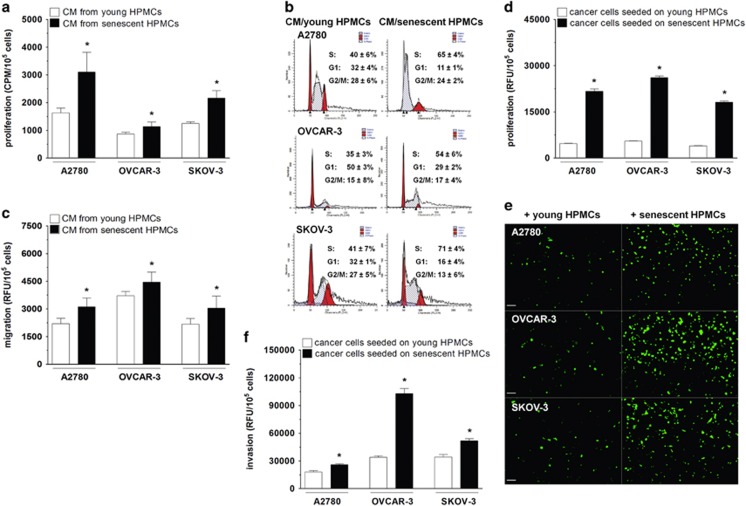
Effect of senescent HPMCs on the progression of ovarian cancer cells *in vitro* ovarian cancer cells were subjected to CM from young and senescent HPMCs, then their proliferation (**a**), distribution in the cell cycle (**b**), and migration (**c**) were measured. In addition, the cancer cells were seeded on top of young and senescent HPMCs in order to examine their proliferation (**d**-**e**) and invasion (**f**). The hatched areas in the histograms shown in panel (**b**) indicate cells in the S phase of the cell cycle. Panel (**e**) shows representative pictures of fluorescence emitted by GFP-transfected cancer cells growing in direct contact with the HPMCs (× 100; bar, 100 *μ*m). The asterisks indicate significant differences as compared with young HPMCs. The experiments were performed in duplicates using HPMCs obtained from 14 different donors. The results are expressed as mean±S.D.

Ovarian cancer cells subjected to conditioned media (CM) generated by senescent HPMCs proliferated more vigorously than their counterparts exposed to CM from young cells (Figure 1a). This effect was accompanied by an increased fraction of cancer cells that were actively replicating their DNA, that is, cells in the S phase of the cell cycle (Figure 1b). Moreover, cancer cells exposed to CM from senescent HPMCs displayed upregulated expression of several genes regulating the course of the mitotic cycle. Importantly, these changes appeared to be a cell line-specific (Table 2).

When it comes to migration, the efficiency of this process towards a chemotactic gradient generated by CM obtained from senescent cells was considerably higher as compared to the effect elicited by CM from the young cells (Figure 1c).

Afterwards, the effect of direct cell-cell contact on cancer cell proliferation and invasion was evaluated. In this respect, the proliferative capacity of green fluorescence protein (GFP)-transfected cancer cells seeded on top of monolayered senescent HPMCs was significantly intensified (Figures 1d and e). A similar intensification was also observed with regard to cancer cell invasion across a layer of senescent cells resting on Matrigel (Figure 1f).

### Senescent HPMCs promote the intraperitoneal development of ovarian tumors in mice

**Figure 2:**
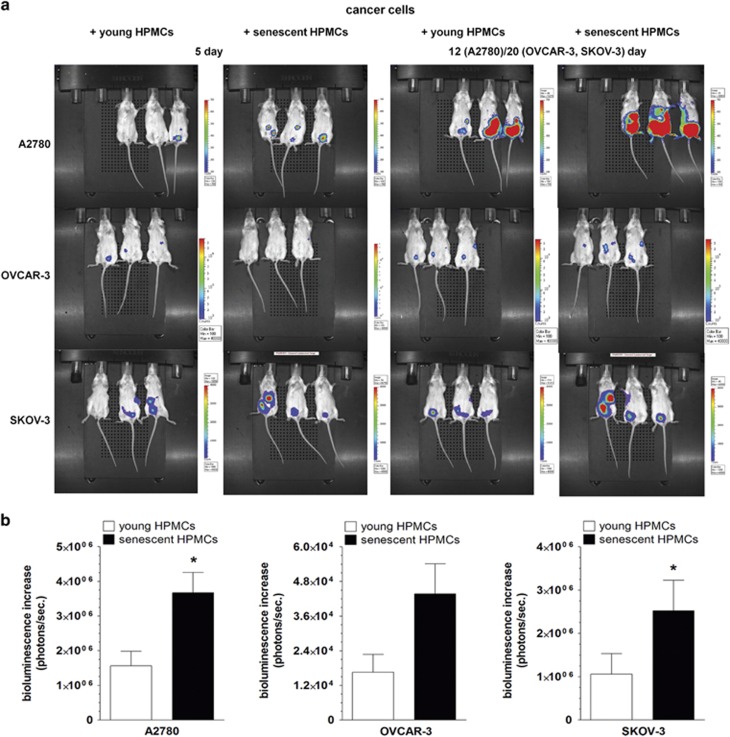
Examination of the intraperitoneal development of ovarian tumors upon i.p. injection of ovarian cancer cells together with young or senescent HPMCs. Representative images showing bioluminescence intensity recorded 5 and 12 (A2780) or 20 (OVCAR-3, SKOV-3) days after cell implantation (**a**). The dynamics of xenograft development, estimated according to the difference between the highest bioluminescence intensity recorded throughout the experiment and the initial value, were recorded 5 days after cell injection (**b**). The asterisks indicate a significant difference as compared with xenografts established in the presence of young HPMCs. Experiments were performed on seven animals per group with HPMCs established from six different donors. The results are expressed as mean±S.D.

Tumors that had formed *in vivo* upon the co-injection i.p. of ovarian cancer cells with senescent HPMCs progressed at higher dynamics than those in which the cancer cells were accompanied by young HPMCs. This effect was evident for all three ovarian cancer cell lines studied (Figure 2).

### HPMCs exhibit a senescence-associated secretory phenotype

Senescent HPMCs appeared to uniformly overexpress transcripts for a plethora of agents involved in angiogenesis, inflammation and extracellular matrix remodeling. The same was the case for genes involved in cell cycle inhibition. The expression of transcripts for genes involved in cell cycle progression was diminished (Table 3).

After qPCR analysis was performed, targets whose products were secreted in a soluble form to the environment were quantified in CM. These investigations showed that the production of 12 out of 18 proteins examined by senescent HPMCs was significantly upregulated as compared with their secretion by the young cells (Table 3).

### Mediators of the pro-cancerous activity of senescent HPMCs are cancer cell line-specific

Exogenous forms of agents secreted at an increased level by senescent HPMCs were used to find which of them may be responsible for the ovarian cancer cell-promoting activity of senescent HPMCs. The studies, performed in both dose-response and time-course regimens (see Supplementary files S1–S12), revealed that several of the tested proteins were capable of stimulating cancer cell proliferation and/or migration. The composition of the stimuli appeared to be cancer cell line-specific (Table 4).

**Figure 3:**
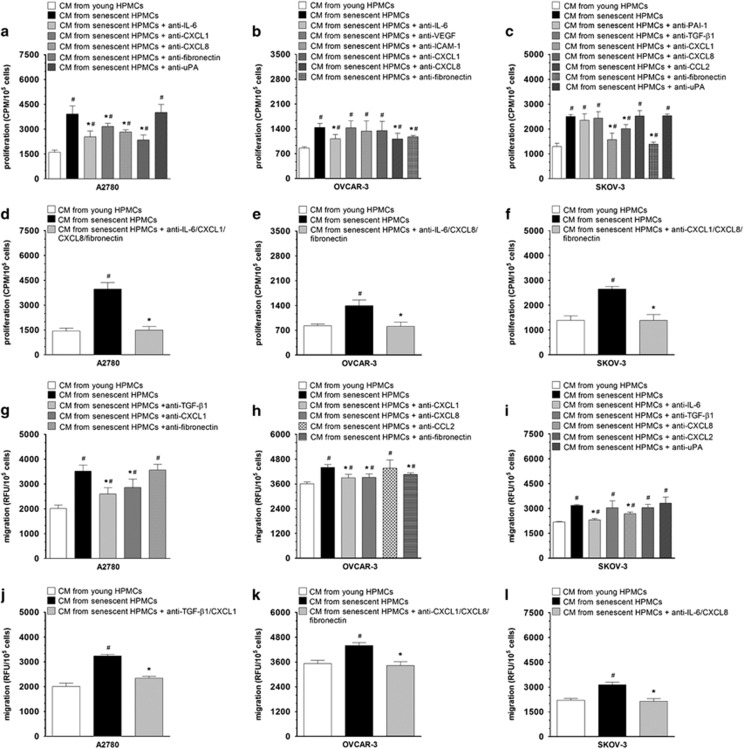
Identification of soluble mediators of the pro-cancerous activity of senescent HPMCs ovarian cancer cell proliferation (**a**-**c**) and migration (**g**-**i**) were investigated in the presence of a CM generated by senescent HPMCs upon its pre-incubation with specific neutralizing antibodies directed against proteins that were initially identified as plausible mediators of increased proliferation and migration of the cancer cells (see Table 3). Because the reduction of the cancer cell progression that was observed in these experiments was only partial, subsequent studies on cancer cell proliferation (**d**-**f**) and migration (**j**-**l**) were conducted in which proteins whose neutralization reduced the stimulatory effect of senescent HPMCs were blocked simultaneously in one sample of the CM. The asterisks indicate significant differences as compared with cells exposed to CM from senescent HPMCs. The hashes indicate significant differences as compared with cells exposed to CM from young HPMCs. The experiments were performed in triplicates using HPMCs obtained from six different donors. The results are expressed as mean±S.D.

Afterwards, cancer cell proliferation and migration were tested again, in the presence of CM from senescent HPMCs that were pre-incubated with specific antibodies or agents directed against the identified proteins. These studies excluded some proteins from the list of senescent HPMC activity mediators when their neutralization in CM failed to reduce the progression of the cancer cells (Figures 3a–c and g–i).

Because the reduction of cancer cell proliferation and migration upon the neutralization of individual proteins was only partial, subsequent experiments were performed in which proteins whose neutralization yielded a significant reduction in cancer cell progression were blocked simultaneously in one sample of the CM. These tests allowed to establish that the growth-promoting activity of senescent HPMCs was related to the hypersecretion of CXCL1, CXCL8, IL-6, and fibronectin (for A2780 cells); CXCL8, IL-6, and fibronectin (for OVCAR-3 cells); and CXCL1, CXCL8, and fibronectin (for SKOV-3 cells) (Figures 3d–f). As for cancer cell migration, senescent HPMCs stimulated this process via CXCL1 and TGF-*β*1 (in A2780 cultures); CXCL1, CXCL8, and fibronectin in OVCAR-3 cells; and CXCL8 and IL-6 in SKOV-3 cells (Figures 3j–l).

### Neutralization of p38 MAPK retards the development of the senescence phenotype in HPMCs and prevents their pro-cancerous activity

**Figure 4:**
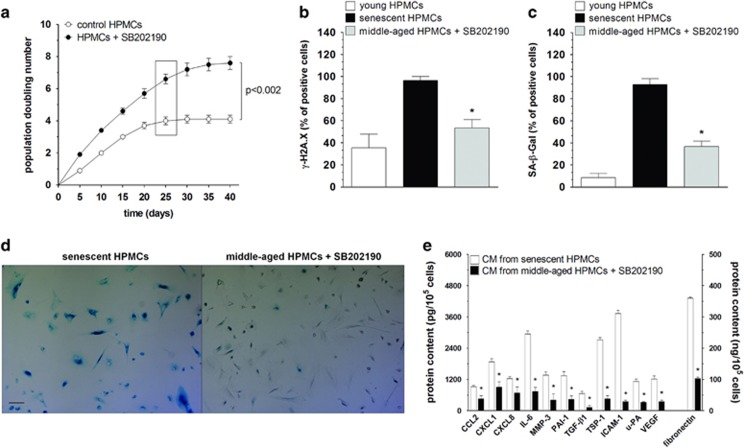
Role of p38 MAPK as a mediator of HPMC senescence replication (**a**), the level of *γ*-H2A.X foci (**b**) and SA-*β*-Gal (**c**-**d**), and the secretion of proteins (**e**) were compared in HPMCs undergoing senescence in standard culture conditions (control cells) and in cells exposed to the p38 MAPK inhibitor – SB202190. The frame located in panel (**a**) indicates a time point at which HPMCs propagated in standard conditions reached senescence. The results shown in panels (**b**, **c**, **d**, and **e**) refer to this moment. Panel (**d**) shows representative results of staining against SA-*β*-Gal (positive cells are green; × 100; bar, 100 *μ*m). The asterisks indicate significant differences as compared with senescent HPMCs. The experiments were performed by using HPMCs obtained from 12 different donors. The results are expressed as mean±S.D.

We designed experiments to verify if blockage of p38 MAPK (using SB202190) may slow down the development of senescence phenotype in HPMCs and prevent the stimulatory effect of senescent cells on the intraperitoneal progression of ovarian tumors *in vivo*. Experiments showed that when the control HPMCs became senescent, cells with inhibited p38 MAPK still proliferated vigorously and displayed a reduced expression of SA-*β*-Gal and *γ*-H2A.X. This coincided with a decreased secretion of proteins overproduced by senescent HPMCs (Figure 4).

**Figure 5:**
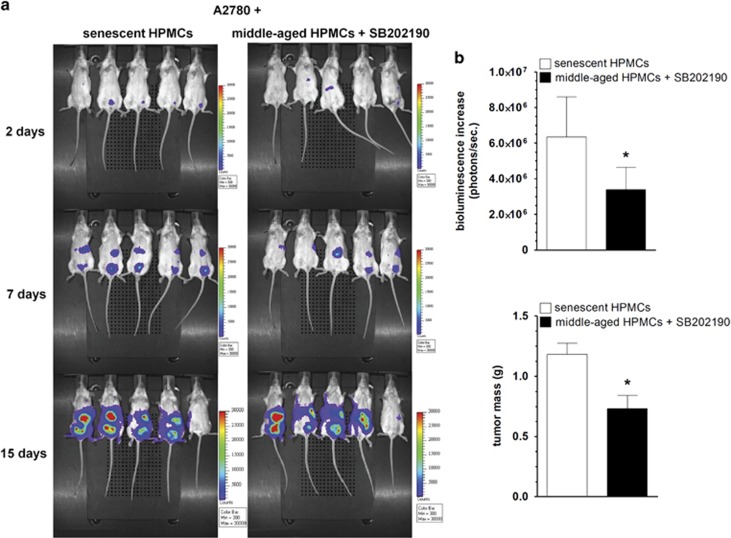
Effect of HPMC rejuvenation by p38 MAPK inhibition on the intraperitoneal development of ovarian tumors in mice. Representative pictures showing bioluminescence intensity of A2780 cells co-injected i.p. with senescent HPMCs or with middle-aged HPMCs treated with SB202190 (**a**). The dynamics of xenograft development, estimated according to the difference between the highest bioluminescence intensity recorded throughout the experiment and the initial value, were recorded 2 days after cell implantation (**b**). Comparison of masses of tumors excised from a mouse peritoneum at the end of the experiment (**c**). The asterisks indicate a significant difference as compared with xenografts established in the presence of senescent HPMCs. Experiments were performed on five animals per group with HPMCs established from five different donors. The results are expressed as mean±S.D.

Further experiments using SCID mice showed that the dynamics of xenograft development and tumor mass were significantly decreased when the A2780 cells (used as a representative of the ovarian cancer model) were accompanied by HPMCs rejuvenated by the inhibition of p38 MAPK (Figure 5).

### Senescent HPMCs are present nearby cancerous tissue in the human peritoneum

**Figure 6:**
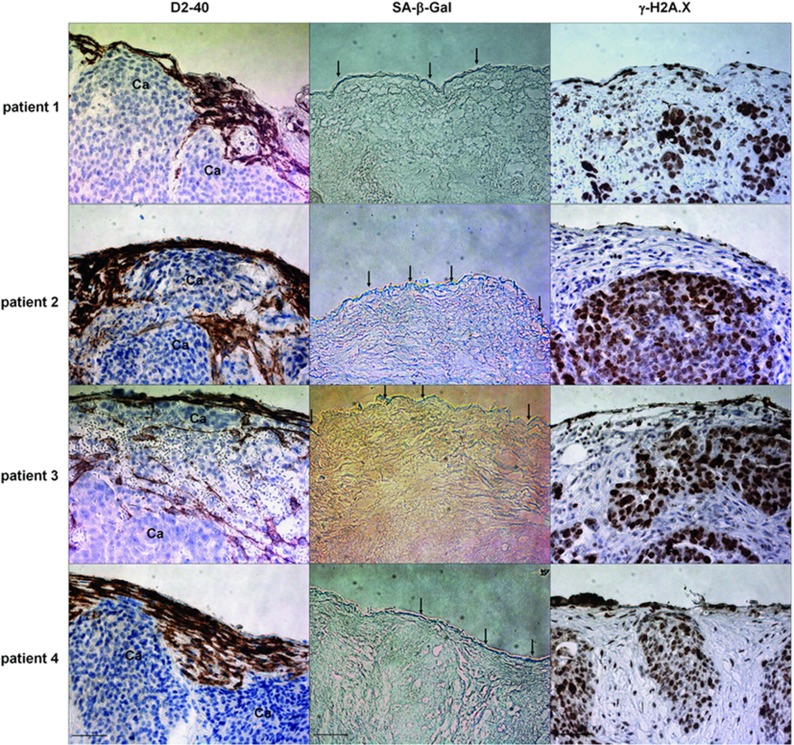
Presence of senescent HPMCs in proximity to cancerous tissue in tumors excised from the peritoneal cavity of patients with ovarian cancer HPMCs were identified according to a positive (brown) reaction against D2-40 antigen. Senescent cells were identified according to the expression (green) of SA-*β*-Gal and a positive reaction (brown) against *γ*-H2A.X. The pictures shown in this figure are representative examples obtained from 4 out of 11 patients examined. Arrows indicate exemplary positive reactions against SA-*β*-Gal. CA – cancerous tissue. Magnification × 400; bar, 50 *μ*m

Analysis of tumors excised from patients with ovarian cancer revealed that HPMCs (identified according to immunostaining for the D2-40 antigen) were consistently present in the direct neighborhood of the cancer cells, and that majority of these cells was positive for SA-*β*-Gal and *γ*-H2A.X (Figure 6).

## Discussion

Existing literature, though pointing to the age dependency of ovarian cancer occurrence^1^ and aggressiveness,^2^ does not explain in a satisfactory manner whether peritoneal ovarian cancer metastases are more frequent in elderly patients. To solve this issue, we compared the incidence of peritoneal tumors as a function of aging, by splitting patients arbitrarily into three age groups and objectively, according to their menopause status.^13^ The analysis showed that, regardless of how the patients were divided, women with intraperitoneal cancer were older than those in whom the pathology did not encompass the peritoneum.

Further experiments showed that senescent HPMCs promote, more efficiently than young cells, proliferation, migration, and invasion of three representative lines of ovarian cancer cells *in vitro*. This effect was related to the activity of soluble agents released by these cells to the environment as well as to their direct interactions with the cancer cells. The strength of the cell-cell interactions was greater than the impact of the soluble agents, which resembles the situation described for breast cancer cells and senescent fibroblasts.^15^ It is likely, however, that the prevalence of cell-cell interactions might not be associated with some explicit growth-promoting effect *per se* but rather by two overlapping phenomena, that is, increased ovarian cancer cell adhesion,^11^ and improved proliferation caused by soluble stimuli released by the senescent cells. Cancer-promoting activity of senescent HPMCs was finally confirmed *in vivo* using immunocompromised mice in which these cells remarkably stimulated the progression of intraperitoneal ovarian tumors. Previously, such activity of senescent cells was reported with regard to breast^15^ and lung cancer cells.^16^ It is worthy to note that the pro-cancerous effect of the senescent HPMCs *in vivo* could be theoretically modulated by the activity (e.g., secretome) of mouse peritoneal mesothelium. The possible reciprocal interplay between mouse cells and the human HPMCs and ovarian cancer cells remains to be elucidated.

From a mechanistic point of view, the pro-cancerogenic activity of senescent HPMCs was associated with increased release and paracrine activity of CXCL1, CXCL8, IL-6, fibronectin, and TGF-*β*1. Intriguingly, these proteins appeared to promote the cancer cells in a cell line-specific manner. The first four agents were already known as capable of stimulating cancer cell proliferation and/or migration,^17, 18, 19^ whereas TGF-*β*1 was not linked with such activity. On the other hand, TGF-*β*1 contributes to ovarian cancer cell invasion,^20^ which in combination with its ability to induce epithelial-mesenchymal transition^21^ may imply its engagement in senescent HPMC-mediated intensification of ovarian cancer cell progression.

Significantly, when the development of senescence phenotype in HPMCs was inhibited by the neutralization of p38 MAPK, the prime signaling molecule underlying senescence of HPMCs,^11, 22^ the rejuvenated cells failed to support the development of ovarian tumors in a mouse peritoneum. This result is coherent with findings provided by another group that had evidenced the regulatory role of p38 MAPK in stromal-mediated tumorigenesis *in vivo*.^23^

In the last part of the project, we analyzed whether senescent HPMCs are present in the neighborhood of cancer cells in the peritoneum of patients with ovarian tumors. Study showed that, indeed, SA-*β*-Gal- and *γ*-H2A.X-positive HPMCs are localized in close proximity to the cancer cells, strengthening our theory about their contribution to the intraperitoneal spread of the disease. At the same time, the observation of an intact layer of HPMCs above the tumor mass challenges the previous findings of Iwanicki *et al.*^24^ who showed a lack of HPMCs in tumors attached to peritoneal organs. In this context it should be stressed that HPMCs with biochemical features of senescence accumulate progressively in the peritoneum during aging also in non-cancerous patients.^25^ This may indicate that they create in the peritoneal cavity a niche in which cancer cells encounter hospitable conditions allowing them to efficiently disseminate. This scenario seems to be of special importance in face of findings by Krtolica *et al.*,^15^ who demonstrated that senescent cells can exert their pro-cancerous activity even when their fraction is as small as 10%.

Collectively, our study provides evidence that pro-cancerous activity of senescent HPMCs may underlie increased intraperitoneal progression of ovarian cancer in elderly individuals. Significant reduction of tumor growth upon the rejuvenation of HPMCs via the inhibition of p38 MAPK may shed new light on new therapeutic ways to treat this type of cancer.

## Materials and methods

### Chemicals

Unless otherwise stated, all chemicals and plastics were from Sigma (St. Louis, MO, USA). Recombinant proteins were from R&D Systems (Abingdon, UK). The neutralizing antibodies, UK-356618 and BC 11 hydrobromide were from R&D Systems (the antibody against TSP-1 was from Abcam (Cambridge, UK)). GRGDSP was from Sigma and SB202190 from Cell Signaling Technology (Danvers, MA, USA).

### Patients

The study included clinical data obtained from 111 women with ovarian cancer who were undergoing surgical treatment at the Division of Gynecological Surgery at Poznań University of Medical Sciences (PUMS). The analysis included five histological subtypes of ovarian cancer, that is: serous (*n*=55), undifferentiated (*n*=28), endometrioid (*n*=13), clear-cell (*n*=9), and mucinous (*n*=6). In addition, tumors excised from the peritoneal cavity with a small margin of normal tissue from 11 patients with serous ovarian cancer (stage III) were used. The tumors were fixed in 4% formalin, embedded in paraffin and cut into 3 *μ*m sections. Deparaffinization, rehydration, and epitope retrieval were conducted using Envision Flex Target Retrieval Solution (Dako, Glostrup, Denmark). The cancerous tissue was identified using H+E staining. Mesothelial cells lying in the proximity of cancer cells were identified according to the presence of the D2-40 antigen.^26^ Antigen visualization was performed using Envision Flex (Dako). The study was approved by the bioethics committee at PUMS (consent number 543/14) and all patients gave their informed consent.

### Cell cultures

HPMCs were isolated by enzymatic digestion of omentum, obtained from 18 patients undergoing abdominal surgery. The age of the donors ranged from 38 to 46 years old. The cells were propagated as described by Mikula-Pietrasik *et al.*^27^ The study was approved by the bioethics committee at PUMS (consent number 754/13) and all patients gave their informed consent.

The HPMCs were forced into senescence by serial passaging at 7-day intervals with a fixed seeding density of 3 × 10^4^ cells/cm^2^. Cells from passages 1–2 were treated as ‘young', while those that had failed to increase in number during 4 weeks and stained >70% for SA-*β*-Gal were considered as ‘senescent'. In some experiments, the HPMCs were treated with p38 MAPK inhibitor, SB202190 (10 *μ*M) for 2 h daily until the moment at which their counterparts cultured standard conditions became senescent.

The presence of cellular senescence biomarkers, senescence-associated *β*-galactosidase (SA-*β*-Gal) and histone *γ*-H2A.X foci in the HPMCs was evaluated as described by Mikula-Pietrasik *et al.*^22^ The presence of γ-H2A.X-positive cells in tumors was detected, as described by Xie *et al.*^28^

The ovarian cancer cell lines A2780 and SKOV-3 were purchased from the ECCC (Porton Down, UK), whereas the OVCAR-3 line was obtained from the ATCC (Rockville, MD, USA). The cancer cells were propagated as described by Ksiazek *et al.*^11^

### Determination of cancer cell proliferation, migration, and invasion

Proliferation of cancer cells subjected to HPMC-derived conditioned medium for 72 h was examined in serum-free conditions using the radioisotope method and with the flow cytometry (FACSCalibur with ModFit LT software (Verity Software House, Topsham, ME, USA)), as described by Mikula-Pietrasik *et al.*,^22,29^ respectively. Before the exposure to the conditioned medium, cancer cells were growth synchronized in serum-free medium for 48 h, which guaranteed that the three lines of cells characterized by different proliferative potential will emerge from the arrest with the same efficiency. In the case of cell-cell interaction analysis, proliferation of cancer cells seeded on top of monolayered HPMCs was estimated using a fluorescence method upon the cancer cell transfection with the plasmid for the GFP. The intensity of fluorescence emitted by the cancer cells was recorded using a Wallac Victor 2 spectrofluorometer (Perkin-Elmer, Massachusetts, USA) at 435 nm excitation and 535 nm emission wavelengths, respectively. Simultaneously, GFP fluorescence was visualized using the Zeiss Axio Observer D1 fluorescent microscope (Carl-Zeiss, Jena, Germany).

Cancer cell migration towards a chemotactic gradient generated by young or senescent HPMCs was analyzed using Transwell inserts (Costar, Inc., NY, USA), as described by Mikula-Pietrasik *et al.*^30^ Analysis of cancer cell invasion across monolayered young or senescent HPMCs lying on Matrigel was performed using the BD BioCoat Tumor Invasion Chamber (BD Biosciences, Bedford, MA, USA), as per manufacturer's instructions.

### Intervention studies

In some experiments, ovarian cancer cell proliferation and migration were examined in response to recombinant forms of human CCL2, CXCL1, CXCL8, ICAM-1, IL-6, PAI-1, TGF-*β*1, VEGF, MMP-3, uPA, TSP-1, and fibronectin. In the dose-response experiments, the cancer cells were exposed to these proteins used in the range of concentrations 0.1-10 ng/ml (100-1000 ng/ml for fibronectin) for 6 h at 37°C.

Moreover, cancer cell proliferation and migration were tested in response to conditioned medium from senescent HPMCs which was pre-incubated (for 4 h at 37°C) with neutralizing antibodies against CCL2 (100 *μ*g/ml), PAI-1 (20 *μ*g/ml), TGF-*β*1 (400 ng/ml), IL-6 (200 ng/ml), CXCL1 (10 *μ*g/ml), CXCL8 (20 *μ*g/ml), VEGF (5 *μ*g/ml), ICAM-1 (25 *μ*g/ml), and TSP-1 (5 *μ*g/ml), or with UK-356618 (5 nm) and BC 11 hydrobromide (10 *μ*M), the inhibitors of MMP-3 and u-PA, respectively. In the same set of experiments, cancer cells were pre-incubated with GRGDSP (10 *μ*M) – a peptide that specifically binds to the fibronectin receptor.

### Analysis of gene expression and protein production

Global gene expression was analyzed using quantitative real-time PCR, as described by Mikula-Pietrasik *et al.*^27^ In order to analyze cell secretome, CM were collected from young and senescent HPMCs, as described by Mikula-Pietrasik *et al.*^12^ Proteins were quantified using appropriate DuoSet Immunoassay Development kits (R&D Systems). The concentration of fibronectin was estimated using the Fibronectin Human ELISA Kit, purchased from Abcam.

### Animal studies

The experiments were performed on immunocompromised Scid mice (CB17/ I cr-Prkdc/ I crI coCrl, Charles River, Wilmington, MA, USA) which were injected i.p. with cancer cells mixed with young or senescent HPMCs (both 2 × 10^6^ cells) in 100 *μ*l of PBS, according to the protocol described previously by Mikula-Pietrasik *et al.*^12, 27^ The animals were kept in the experiment for 12 (A2780) or 20 (OVCAR-3, SKOV-3) days and then humanely sacrificed. Non-invasive tumor monitoring with the IVIS Spectrum Imaging System (Caliper Life Sciences, Hopkinton, MA, USA) was performed according to manufacturer's protocol.

In some experiments, the mice were injected i.p. with mixtures of A2780 cells with senescent HPMCs or with HPMCs pre-incubated with SB202190. Aside from the bioluminescence imaging with IVIS for xenograft development quantification, the tumor mass was measured upon the lesion isolation from the peritoneum by experienced surgeon, who also paid particular attention to the identification of micrometastases. The analysis included only those specimens whose cancerous nature was confirmed upon histopathological examination. All procedures on laboratory animals were performed in compliance with the EU Directive 2010/63/EU. The study was approved by the Local Ethics Committee for Experiments on Animals in Poznań (consent number 44/2011).

### Microscopy conditions

Results of microscopic evaluations were visualized under a microscope, Zeiss Axio Observer D1 (Carl-Zeiss, Jena, Germany). The conditions were as follows: magnifications × 100 (objective aperture 0.25) and × 400 (objective aperture 0.6), temperature 37°C, imaging medium: Faramount Aqueous Mounting Medium (Dako), camera: AxioCam MRc5 (Carl-Zeiss). For image acquisition and processing (linear contrast and brightness adjustments), AxioVision v.4.6 software (Carl-Zeiss) was used.

### Statistics

Statistical analysis was performed using GraphPad Prism 5.00 (GraphPad Software, San Diego, CA, USA). The means were compared with the Wilcoxon and Mann-Whitney tests. Repeated measures analysis of variance with the Newman-Keuls test as a *post-hoc* test were used when appropriate. The results were expressed as means±S.D. Differences with a *P*-value<0.05 were considered to be statistically significant.

## Figures and Tables

**Table 1:** Effect of aging on the intraperitoneal spread of ovarian cancer

**Age group**	**FIGO I-II**	**FIGO III-IV**
⩽ 39 years	5/8 (63)	3/8 (37)
40-59 years	21/63 (33)	42/63 (67)
⩾ 60 years	12/40 (30)	28/40 (70)
		
**Age group**	**FIGO I-II**	**FIGO III-IV**
⩽ 51 years	19/38 (50)	19/38 (50)
⩾ 51 years	19/73 (26)	54/73 (74)

Patients were divided according to their age at diagnosis. The upper part of the table shows the results when the patients were divided into three age groups. The bottom part shows the results when the patients were divided according to their menopause status (⩽ 51 years - before menopause; ⩾ 51 years - after menopause). The values presented in the table indicate as follows: number of patients in a given age group/total number of patients in this age group (percentage of patients)

**Table 2:** Changes in the expression of genes regulating cell cycle progression in ovarian cancer cells exposed to conditioned medium from senescent HPMCs

	**mRNA change (%)**		
**Target**	**A2780**	**OVCAR-3**	**SKOV-3**
Cyclin A1	98±5	103±11	193±2*
Cyclin A2	233±11*	161±4*	98±7
Cyclin B1	340±22*	189±12*	156±4*
Cyclin D1	163±8*	89±34	100±2
Cyclin E2	196±8*	238±6*	246±1*
Cyclin G2	104±22	196±8*	199±8*
Cell division cycle 2	92±12	313±5*	99±1
Ki67	211±12*	98±8	246±6*
HGF	99±3	101±2	166±4*

The results derive from experiments performed with HPMC cultures established from four different patients and are expressed as the percentage of values recorded for cancer cells subjected to conditioned medium from young HPMCs (means±S.D.). Cancer cells were used in duplicates. The calculation of relative change in mRNA was standardized to the GAPDH housekeeping gene. The asterisks indicate significant differences as compared with the cancer cells exposed to the conditioned medium from young HPMCs

**Table 3:** Senescence-associated secretory phenotype in primary HPMCs

**Function**	**Target**	**mRNA change (%** ***versus*** **young)**	**Protein change (%** ***versus*** **young)**
*Cell cycle transition*	Cyclin A1	23±5*	n.m.
	Cyclin D1	145±12*	n.m.
	Cyclin E	162±3*	n.m.
	PCNA	22±6*	n.m.
	p16(INK4a)	333±21*	n.m.
			
*Angiogenesis*	CXCL1	211±22*	432±31*
	CXCL8	166±13*	186±12*
	CXCL12	199±33*	99±4
	FGF2	181±3*	103±9
	FGFBP1	141±16*	n.m.
	HIF-1	366±44*	n.m.
	VEGF	183±11*	211±22*
			
*Inflammation*	CCL2	321±16*	176±14*
	IL-6	511±99*	154±8*
	ICAM-1	222±31*	421±65*
	IL-6R	178±22*	106±9
			
*ECM synthesis and remodeling*	CTGF	133±6*	n.m.
	Fibronectin	254±21*	216±31*
	MMP-3	211±11*	317±23*
	TGF-*β*1	131±4*	264±11*
	TIMP-1	102±8	103±10
	TIMP-2	98±6	111±18
	TSP-1	421±13*	144±11*
	PAI-1	186±21*	241±13*
	t-PA	176±12*	n.m.
	u-PA	554±65*	188±34*

The results derive from experiments performed with HPMC cultures established from 8 (mRNA) and 22 (protein) different patients and are expressed as the percentage of values recorded for young HPMCs (means±S.D.). The calculation of relative change in mRNA was standardized to the GAPDH housekeeping gene. The asterisks indicate significant differences as compared with young HPMCs. n.m. – not measured

**Table 4:** Proteins identified as possible mediators of increased proliferation and migration of ovarian cancer cells exposed to CM from senescent HPMCs

**Recombinant protein**	**Proliferation**			**Migration**		
	**A2780**	**OVCAR-3**	**SKOV-3**	**A2780**	**OVCAR-3**	**SKOV-3**
CCL2			┼		┼	┼
CXCL1	┼	┼	┼	┼	┼	
CXCL8	┼	┼	┼		┼	┼
Fibronectin	┼	┼	┼	┼	┼	
IL-6	┼	┼				┼
MMP-3						
PAI-1			┼			
TGF-*β*1			┼	┼		┼
TSP-1						
ICAM-1		┼				
uPA	┼		┼			┼
VEGF		┼				

Recombinant forms of proteins were added to standard growth medium in a range of concentrations corresponding to their level in the samples of CM obtained from senescent HPMCs. The ┼ mark indicates that an agent was found to stimulate proliferation or migration of ovarian cancer cells in a time- and/or dose-dependent manner. For detailed information regarding concentrations and time intervals at which given proteins were capable of promoting cancer cells, please refer to the Supplementary data (Supplementary Figures S1-S12)

## Supplementary Material

Supplementary Information Summary
