# Supplementary material for: Senescent peritoneal mesothelium creates a niche for ovarian cancer metastases
Source: Cell Death Dis. 2016 Dec 29;7(12):e2565–. doi: 10.1038/cddis.2016.417 (PMC5261005; doi:10.1038/cddis.2016.417)
Supplement: Supplementary Information Summary [file cddis2016417x1.docx]

Effect of exogenous, recombinant forms of CCL2/MCP-1 (Fig. S1), CXCL1/GRO-1 (Fig. S2), CXCL8/IL-8 (Fig. S3), IL-6 (Fig. S4), MMP-3 (Fig. S5), PAI-1 (Fig. S6), TGF-β1 (Fig. S7), TSP-1 (Fig. S8), sICAM-1 (Fig. S9), uPA (Fig. S10), VEGF (Fig. S11), and fibronectin (Fig. S12) on proliferation and migration of ovarian cancer cells A2780 (A, B), OVCAR-3 (C, D), and SKOV-3 (D, E).
