# Supplementary material for: Senescent peritoneal mesothelium creates a niche for ovarian cancer metastases
Source: Cell Death Dis. 2016 Dec 29;7(12):e2565–. doi: 10.1038/cddis.2016.417 (PMC5261005; doi:10.1038/cddis.2016.417)
Supplement: Supplementary Information [file cddis2016417x2.pdf]

Fig.S1

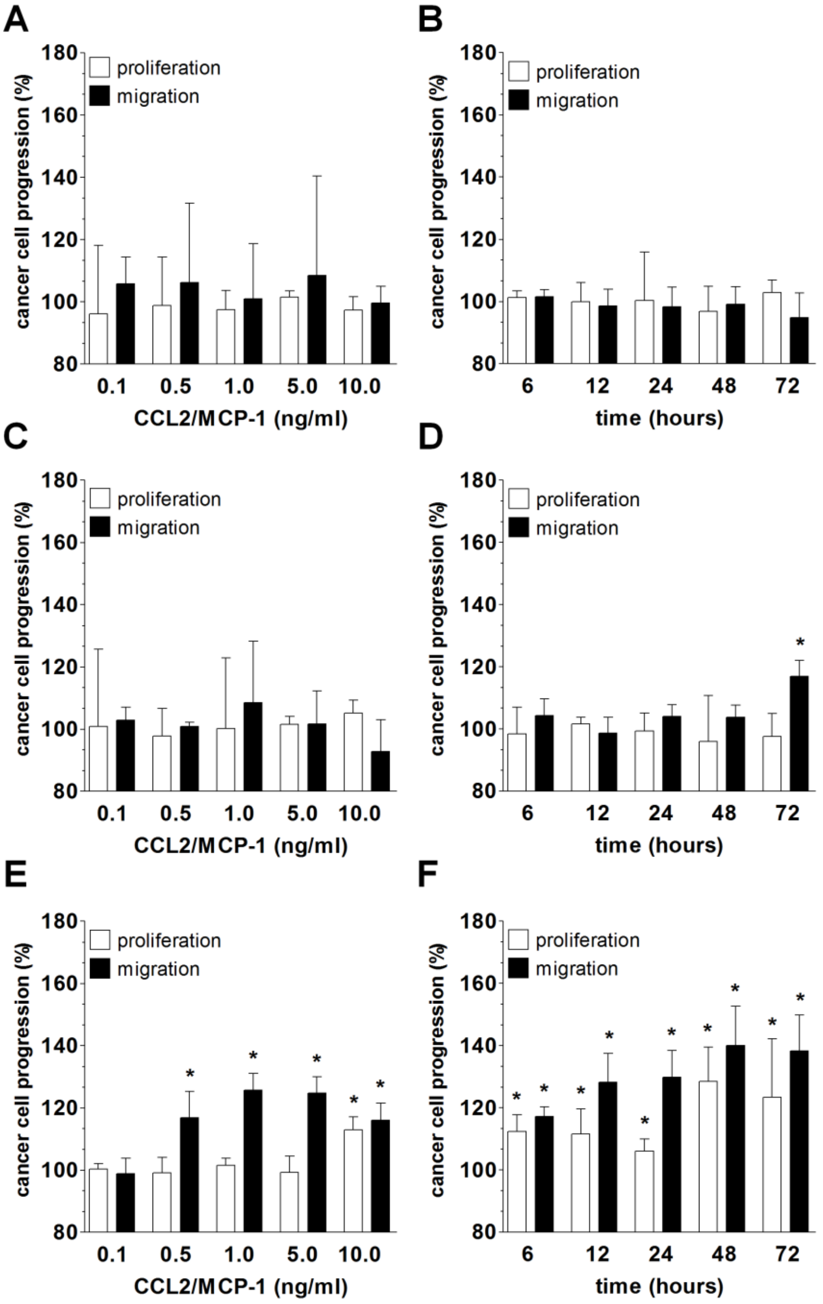

Figure S1

Effect of exogenous, recombinant form of CCL2/MCP-1 on proliferation and migration of ovarian cancer cells A2780 (A, B), OVCAR-3 (C, D) i SKOV-3 (D, E). Left panel (A, C, E) shows results of dose-response experiments while the right one shows results of time-course studies. The results were analysed using repeated measures analysis of variance (ANOVA) with the Newman-Keuls test as a post-hoc test. The asterisks indicate significant differences ($P<0.05$) as compared with the control, untreated cells (100%). The experiments were performed in hexaplicates. The results are expressed as mean ± SD.

Fig.S2

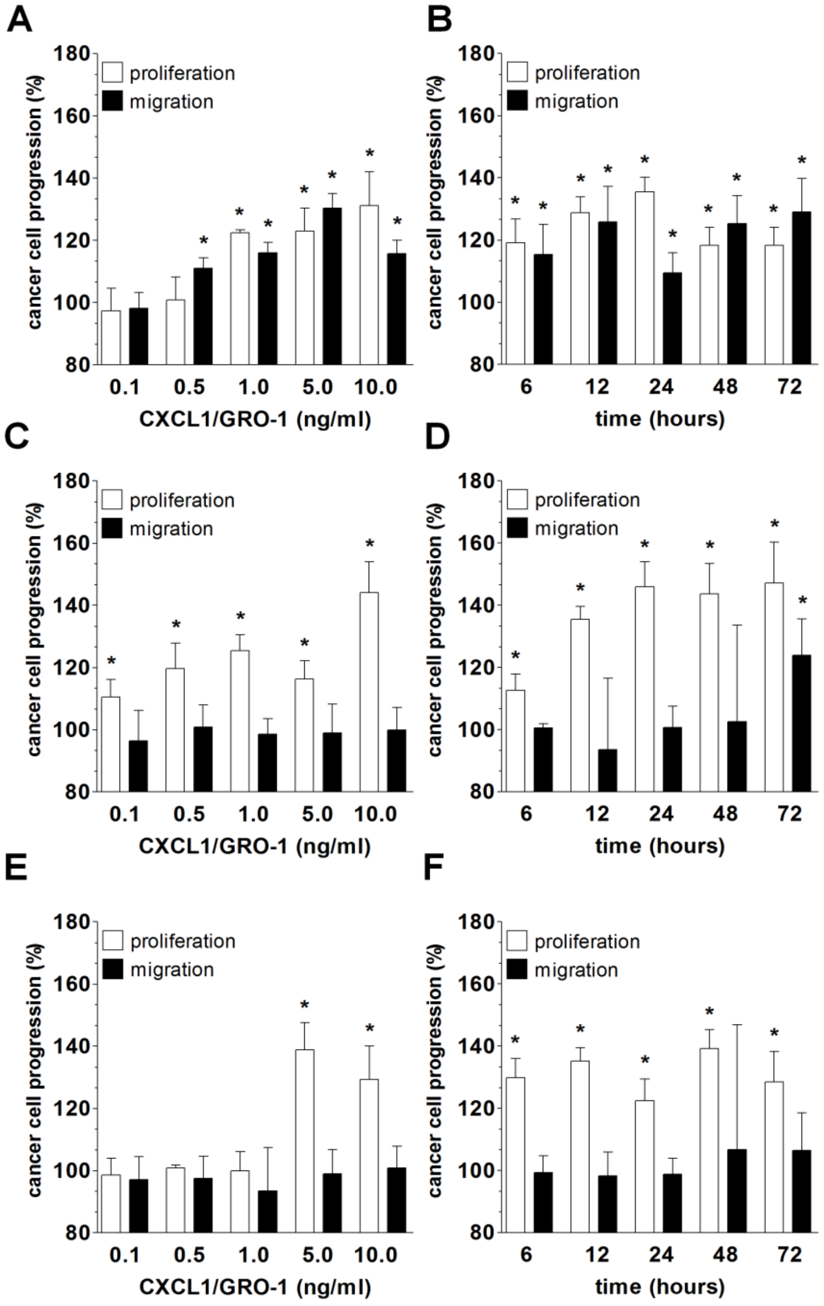

Figure S2

Effect of exogenous, recombinant form of CXCL1/GRO-1 on proliferation and migration of ovarian cancer cells A2780 (A, B), OVCAR-3 (C, D) i SKOV-3 (D, E). Left panel (A, C, E) shows results of dose-response experiments while the right one shows results of time-course studies. The results were analysed using repeated measures analysis of variance (ANOVA) with the Newman-Keuls test as a post-hoc test. The asterisks indicate significant differences ($P<0.05$) as compared with the control, untreated cells (100%). The experiments were performed in hexaplicates. The results are expressed as mean ± SD.

Fig.S3

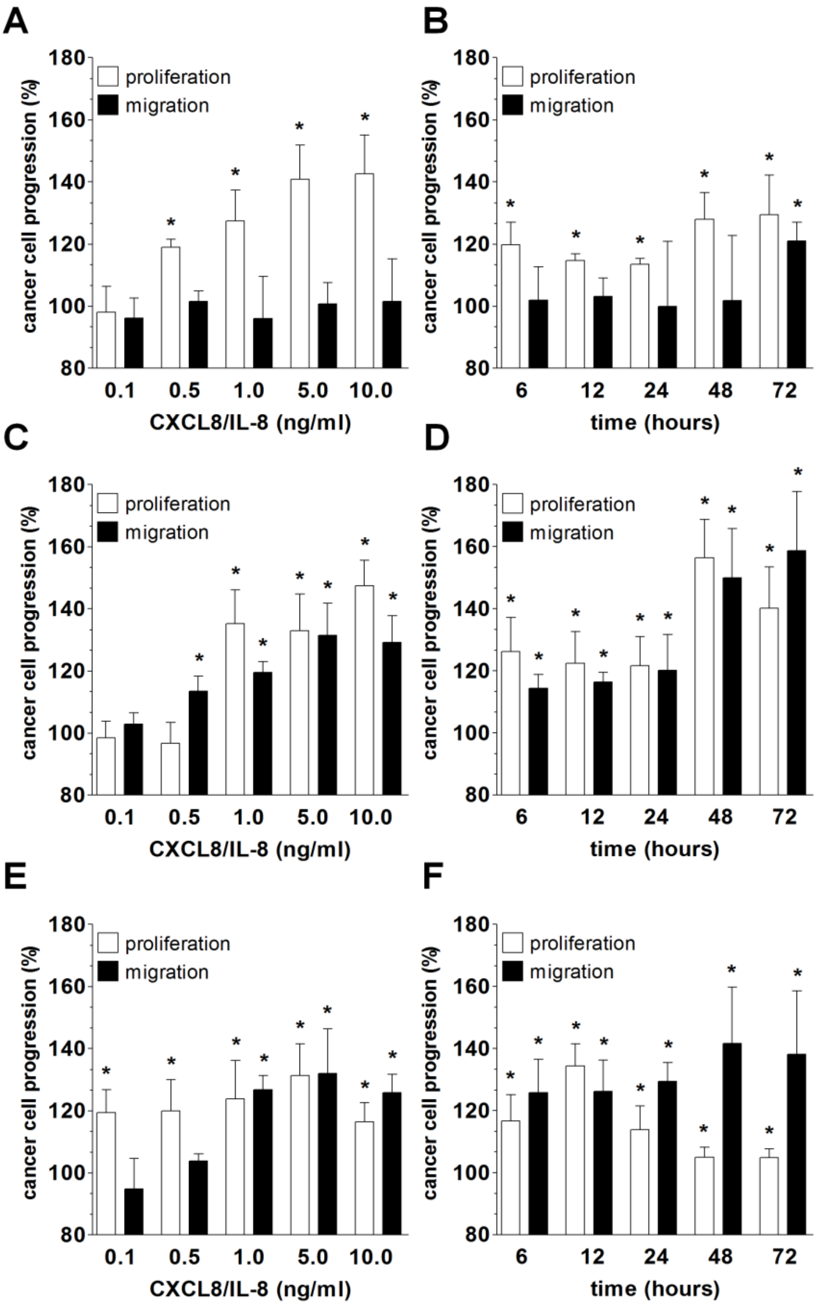

Figure S3
Effect of exogenous, recombinant form of CXCL8/IL-8 on proliferation and migration of ovarian cancer cells A2780 (A, B), OVCAR-3 (C, D) i SKOV-3 (D, E). Left panel (A, C, E) shows results of dose-response experiments while the right one shows results of time-course studies. The results were analysed using repeated measures analysis of variance (ANOVA) with the Newman-Keuls test as a post-hoc test. The asterisks indicate significant differences ($P<0.05$) as compared with the control, untreated cells (100%). The experiments were performed in hexaplicates. The results are expressed as mean ± SD.

Fig.S4

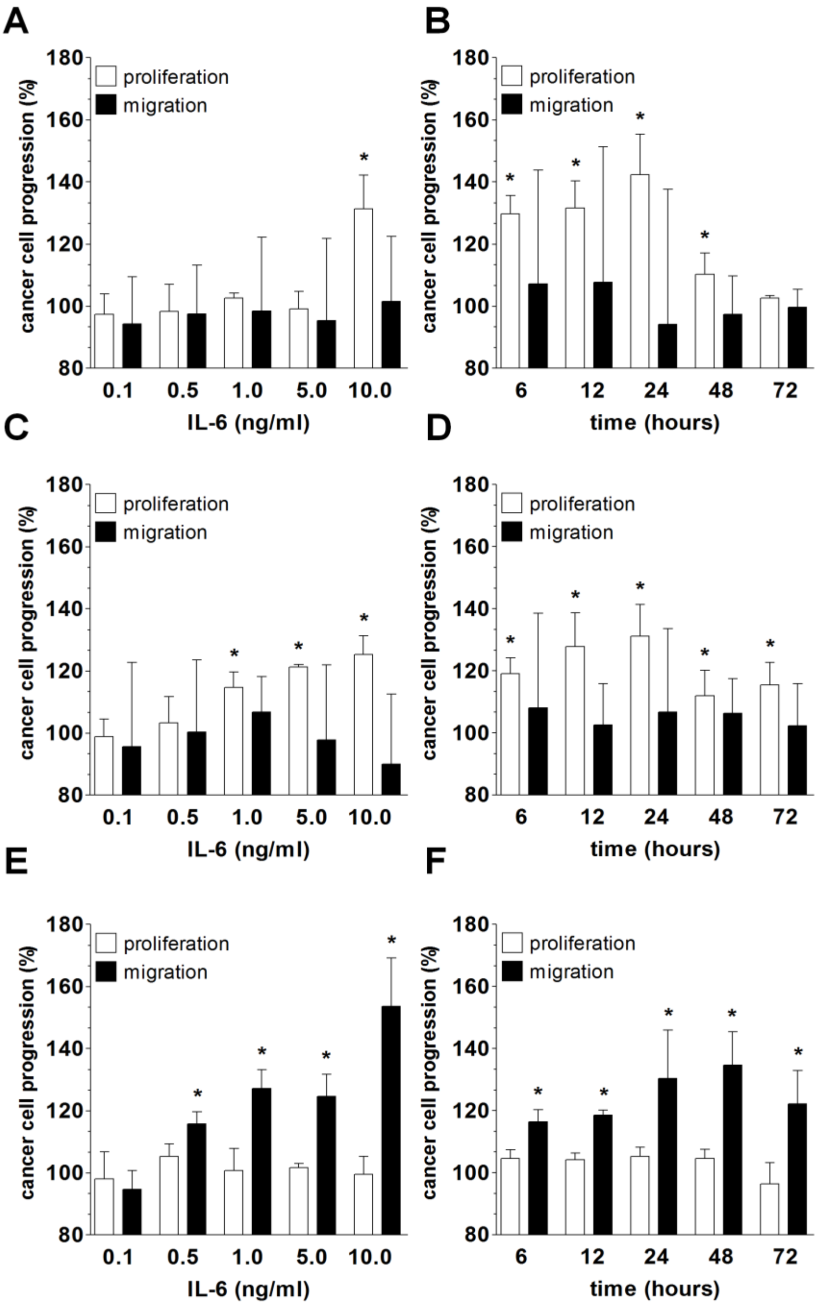

Figure S4

Effect of exogenous, recombinant form of IL-6 on proliferation and migration of ovarian cancer cells A2780 (A, B), OVCAR-3 (C, D) i SKOV-3 (D, E). Left panel (A, C, E) shows results of dose-response experiments while the right one shows results of time-course studies. The results were analysed using repeated measures analysis of variance (ANOVA) with the Newman-Keuls test as a post-hoc test. The asterisks indicate significant differences (P<0.05) as compared with the control, untreated cells (100%). The experiments were performed in hexaplicates. The results are expressed as mean ± SD.

Fig.S5

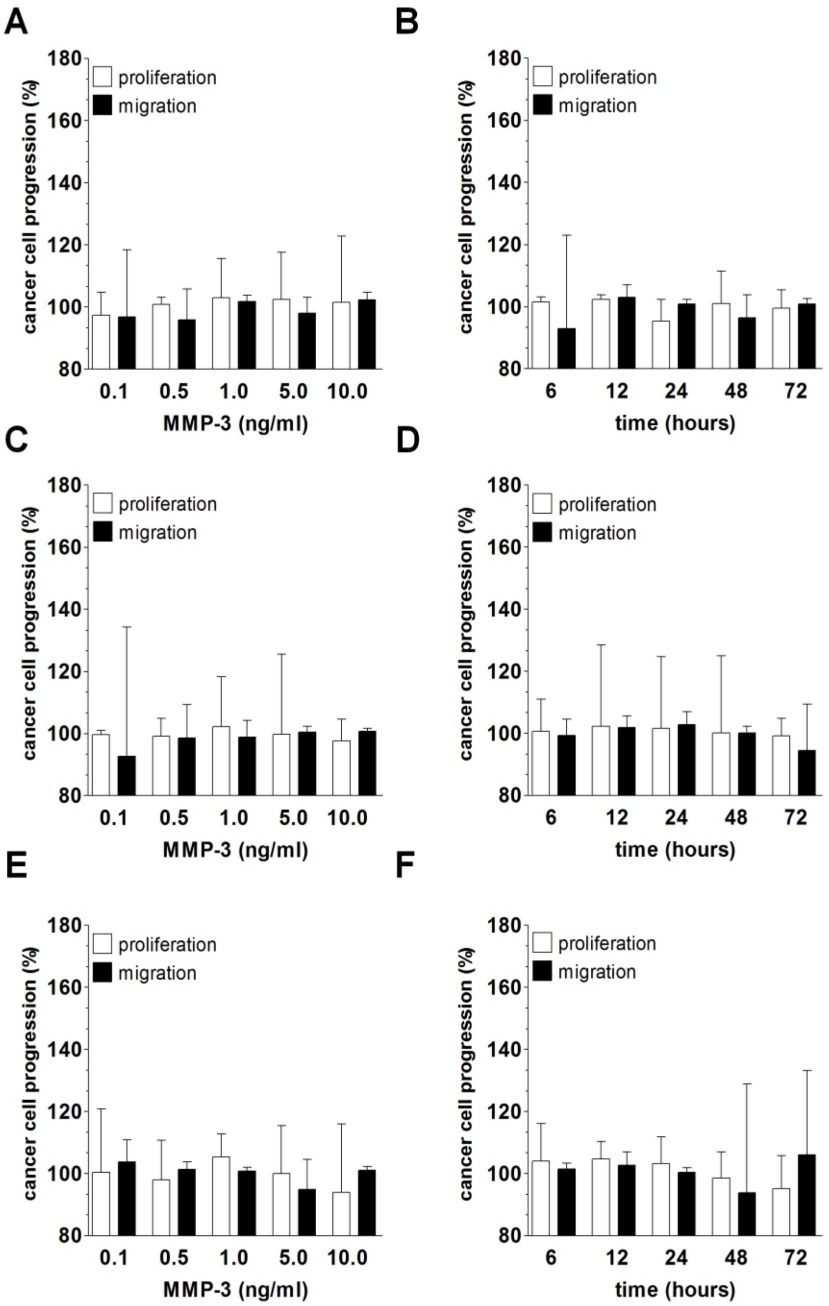

Figure S5
Effect of exogenous, recombinant form of MMP-3 on proliferation and migration of ovarian cancer cells A2780 (A, B), OVCAR-3 (C, D) i SKOV-3 (D, E). Left panel (A, C, E) shows results of dose-response experiments while the right one shows results of time-course studies. The results were analysed using repeated measures analysis of variance (ANOVA) with the Newman-Keuls test as a post-hoc test. The asterisks indicate significant differences ($P<0.05$) as compared with the control, untreated cells (100%). The experiments were performed in hexaplicates. The results are expressed as mean ± SD.

Fig.S6

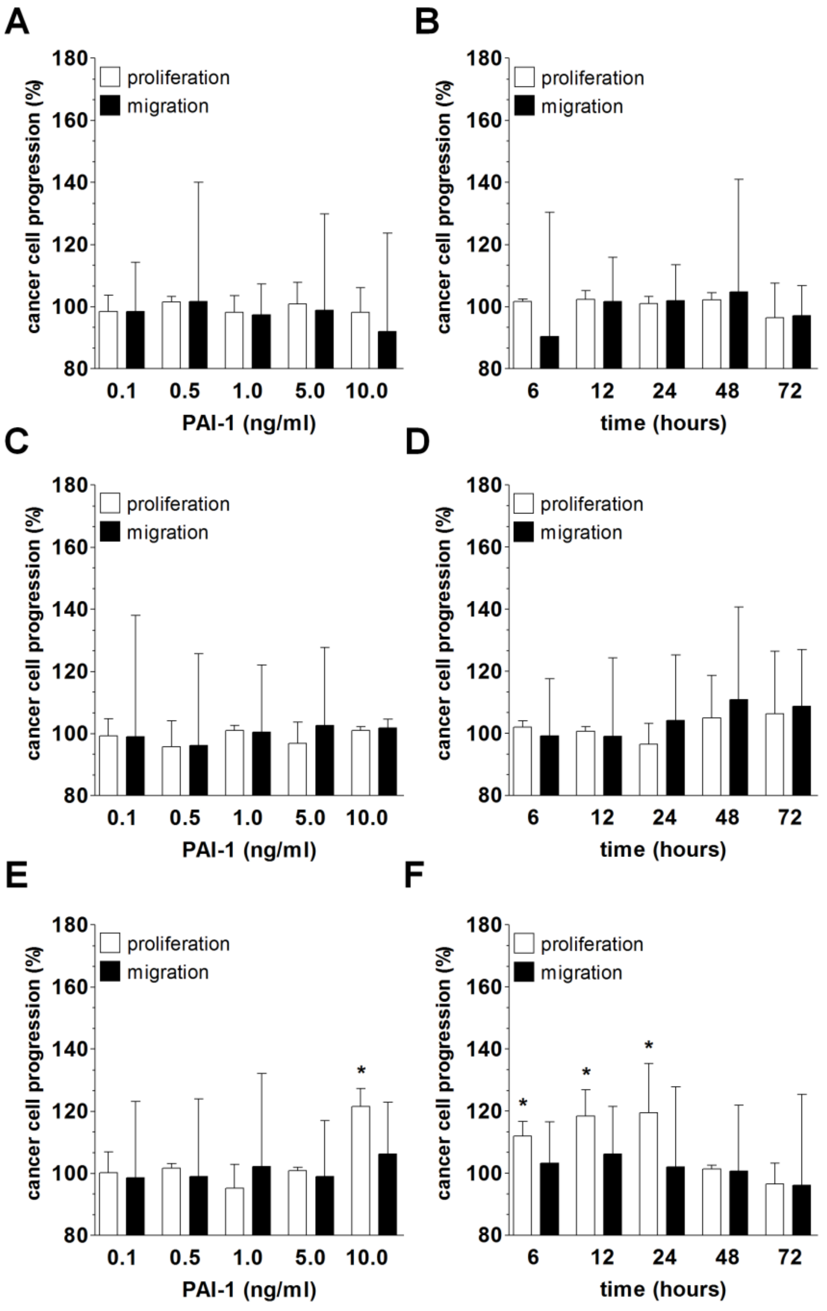

Figure S6
Effect of exogenous, recombinant form of PAI-1 on proliferation and migration of ovarian cancer cells A2780 (A, B), OVCAR-3 (C, D) i SKOV-3 (D, E). Left panel (A, C, E) shows results of dose-response experiments while the right one shows results of time-course studies. The results were analysed using repeated measures analysis of variance (ANOVA) with the Newman-Keuls test as a post-hoc test. The asterisks indicate significant differences ($P<0.05$) as compared with the control, untreated cells (100%). The experiments were performed in hexaplicates. The results are expressed as mean ± SD.

Fig.S7

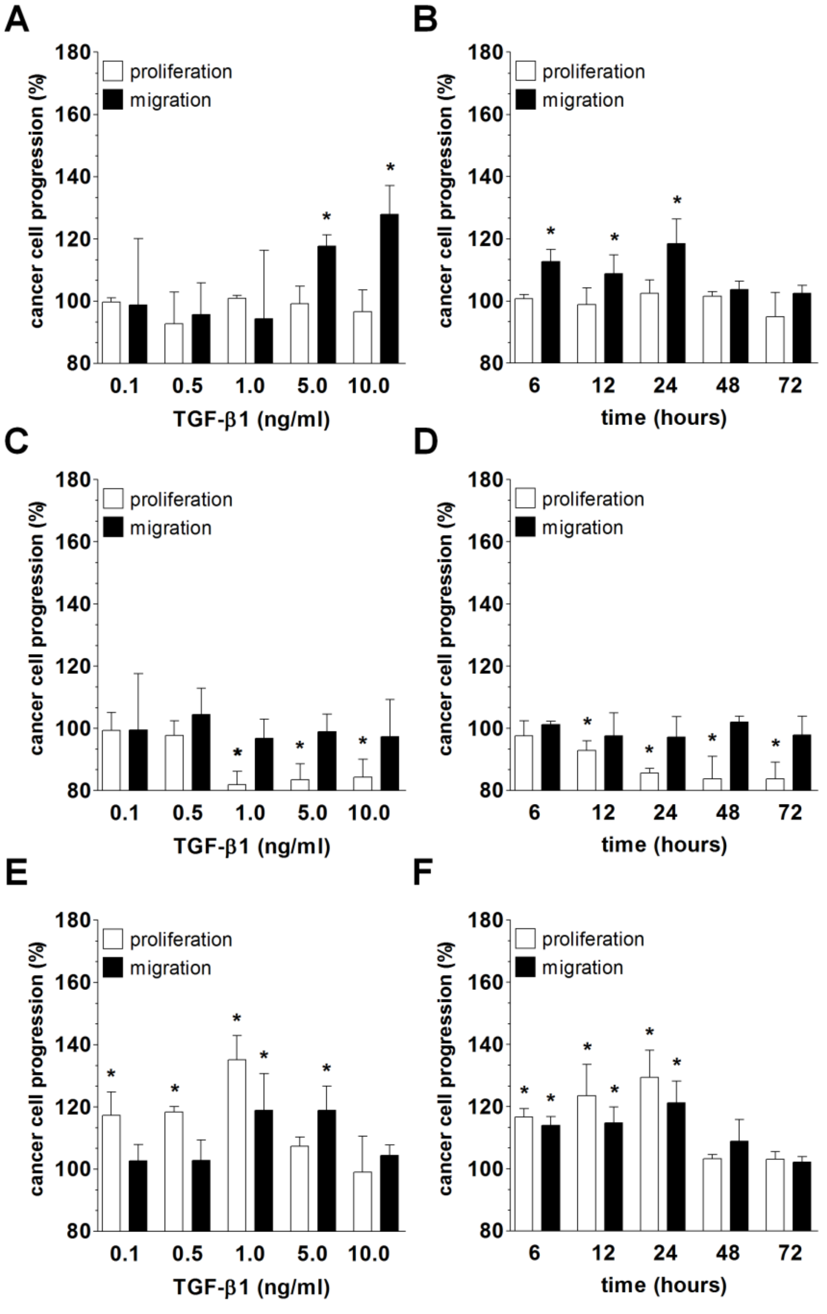

Figure S7
Effect of exogenous, recombinant form of TGF-β1 on proliferation and migration of ovarian cancer cells A2780 (A, B), OVCAR-3 (C, D) i SKOV-3 (D, E). Left panel (A, C, E) shows results of dose-response experiments while the right one shows results of time-course studies. The results were analysed using repeated measures analysis of variance (ANOVA) with the Newman-Keuls test as a post-hoc test. The asterisks indicate significant differences ($P<0.05$) as compared with the control, untreated cells (100%). The experiments were performed in hexaplicates. The results are expressed as mean ± SD.

Fig.S8

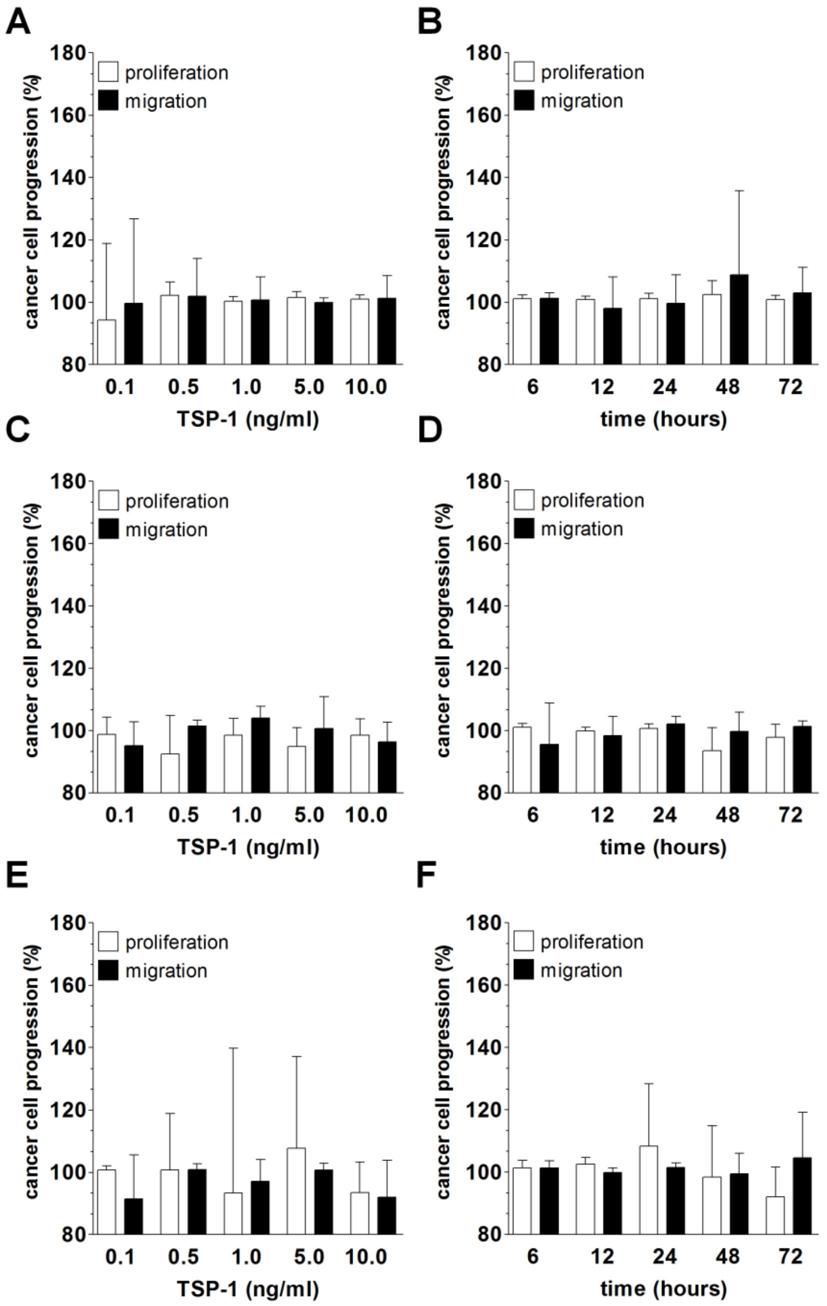

Figure S8
Effect of exogenous, recombinant form of TSP-1 on proliferation and migration of ovarian cancer cells A2780 (A, B), OVCAR-3 (C, D) i SKOV-3 (D, E). Left panel (A, C, E) shows results of dose-response experiments while the right one shows results of time-course studies. The results were analysed using repeated measures analysis of variance (ANOVA) with the Newman-Keuls test as a post-hoc test. The asterisks indicate significant differences ($P<0.05$) as compared with the control, untreated cells (100%). The experiments were performed in hexaplicates. The results are expressed as mean ± SD.

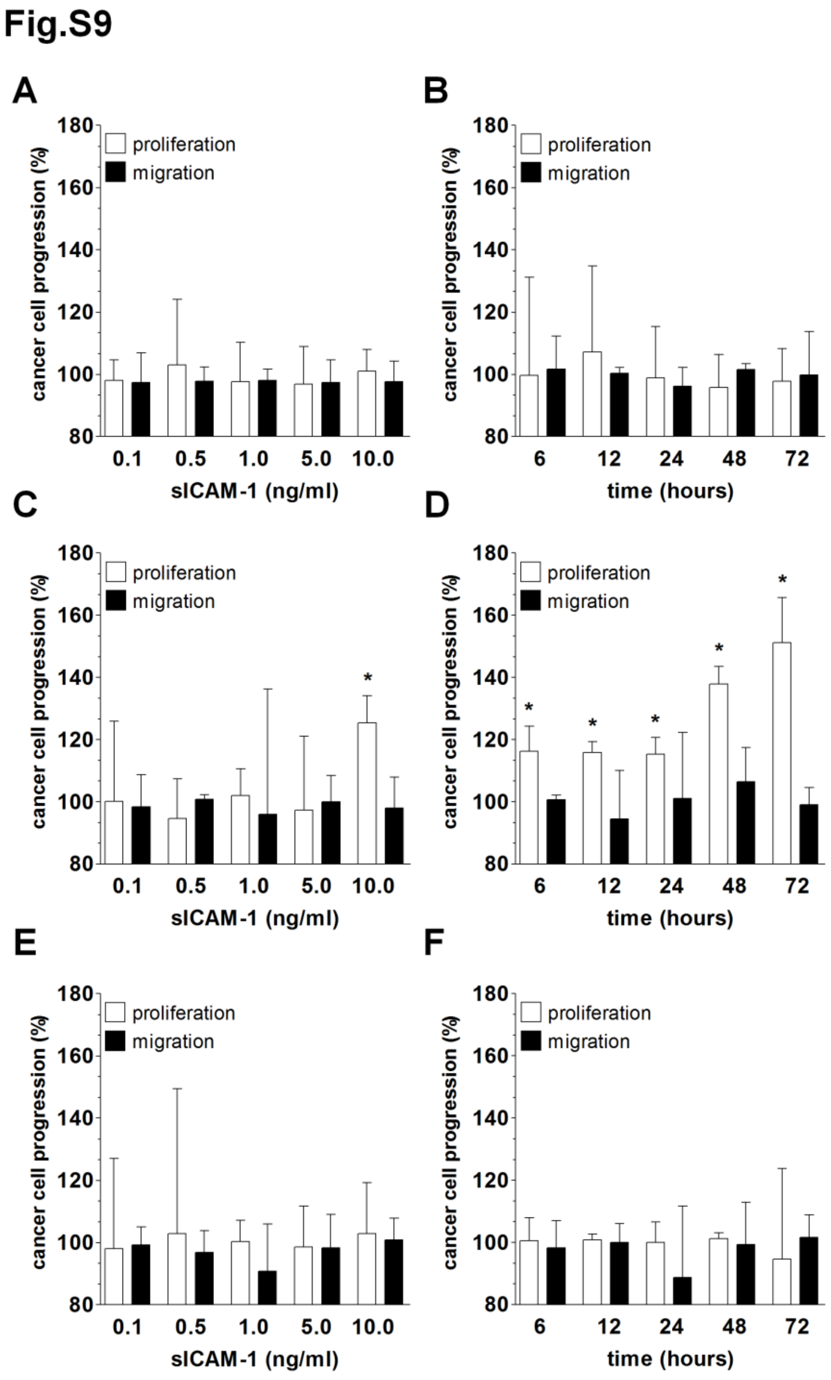

Figure S9

Effect of exogenous, recombinant form of sICAM-1 on proliferation and migration of ovarian cancer cells A2780 (A, B), OVCAR-3 (C, D) i SKOV-3 (D, E). Left panel (A, C, E) shows results of dose-response experiments while the right one shows results of time-course studies. The results were analysed using repeated measures analysis of variance (ANOVA) with the Newman-Keuls test as a post-hoc test. The asterisks indicate significant differences ($P<0.05$) as compared with the control, untreated cells (100%). The experiments were performed in hexaplicates. The results are expressed as mean ± SD.

Fig.S10

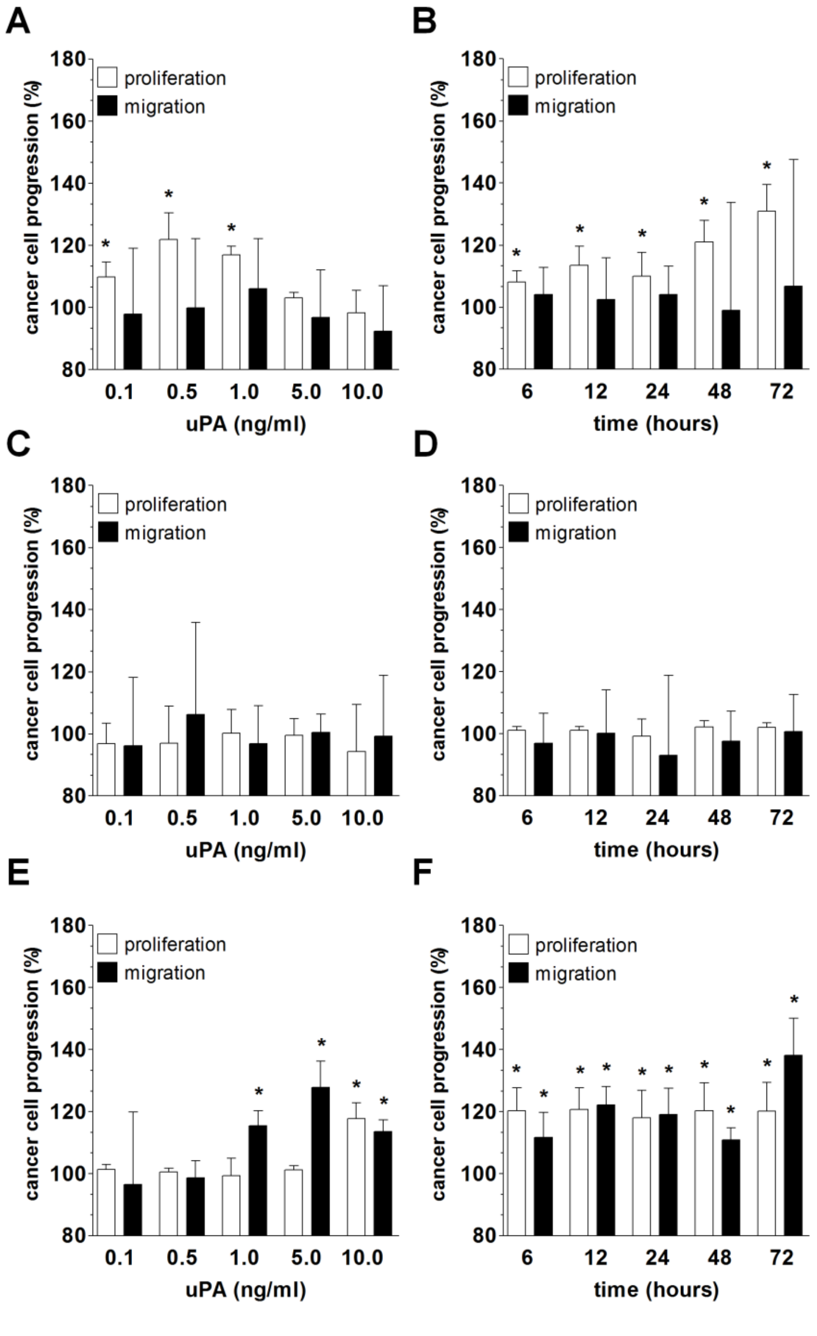

Figure S10
Effect of exogenous, recombinant form of uPA on proliferation and migration of ovarian cancer cells A2780 (A, B), OVCAR-3 (C, D) i SKOV-3 (D, E). Left panel (A, C, E) shows results of dose-response experiments while the right one shows results of time-course studies. The results were analysed using repeated measures analysis of variance (ANOVA) with the Newman-Keuls test as a post-hoc test. The asterisks indicate significant differences (P<0.05) as compared with the control, untreated cells (100%). The experiments were performed in hexaplicates. The results are expressed as mean ± SD.

Fig.S11

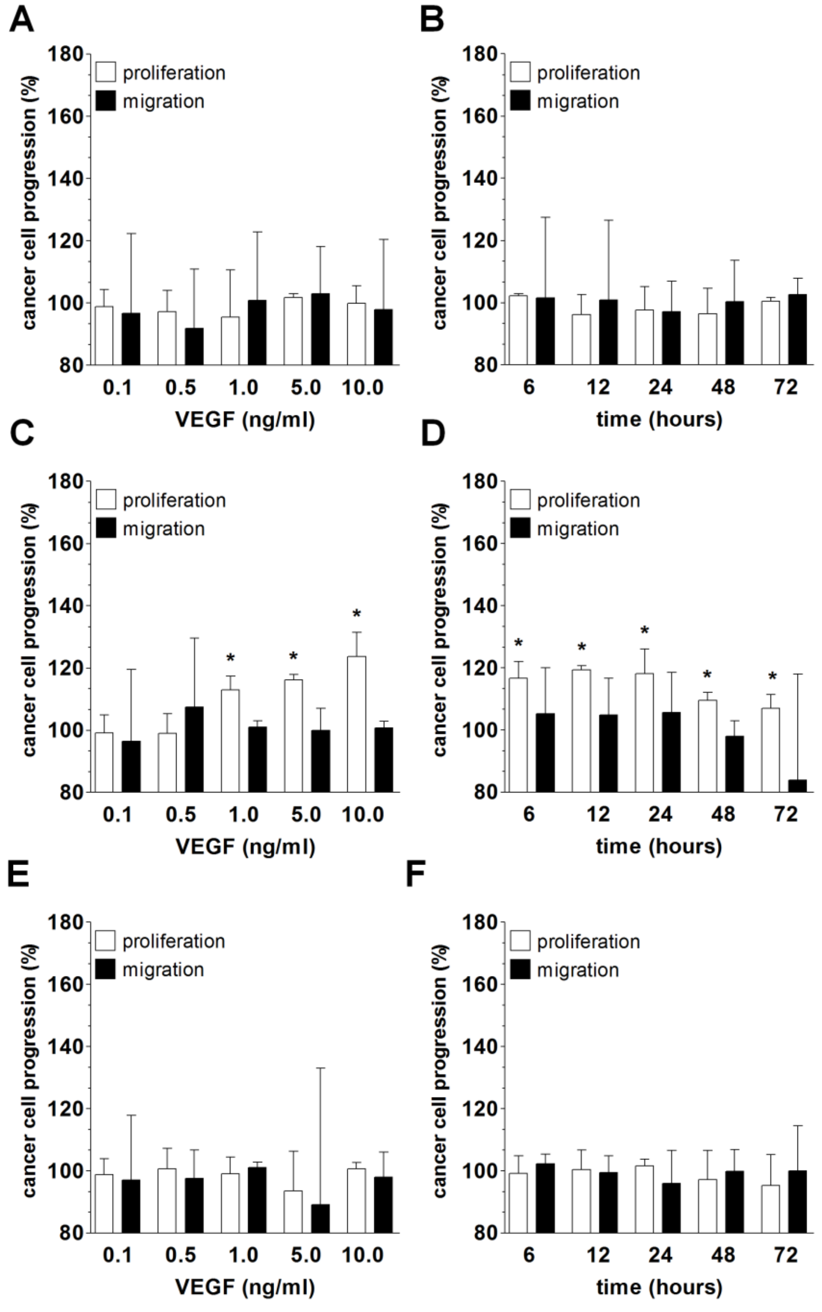

Figure S11
Effect of exogenous, recombinant form of VEGF on proliferation and migration of ovarian cancer cells A2780 (A, B), OVCAR-3 (C, D) i SKOV-3 (D, E). Left panel (A, C, E) shows results of dose-response experiments while the right one shows results of time-course studies. The results were analysed using repeated measures analysis of variance (ANOVA) with the Newman-Keuls test as a post-hoc test. The asterisks indicate significant differences ($P<0.05$) as compared with the control, untreated cells (100%). The experiments were performed in hexaplicates. The results are expressed as mean ± SD.

Fig.S12

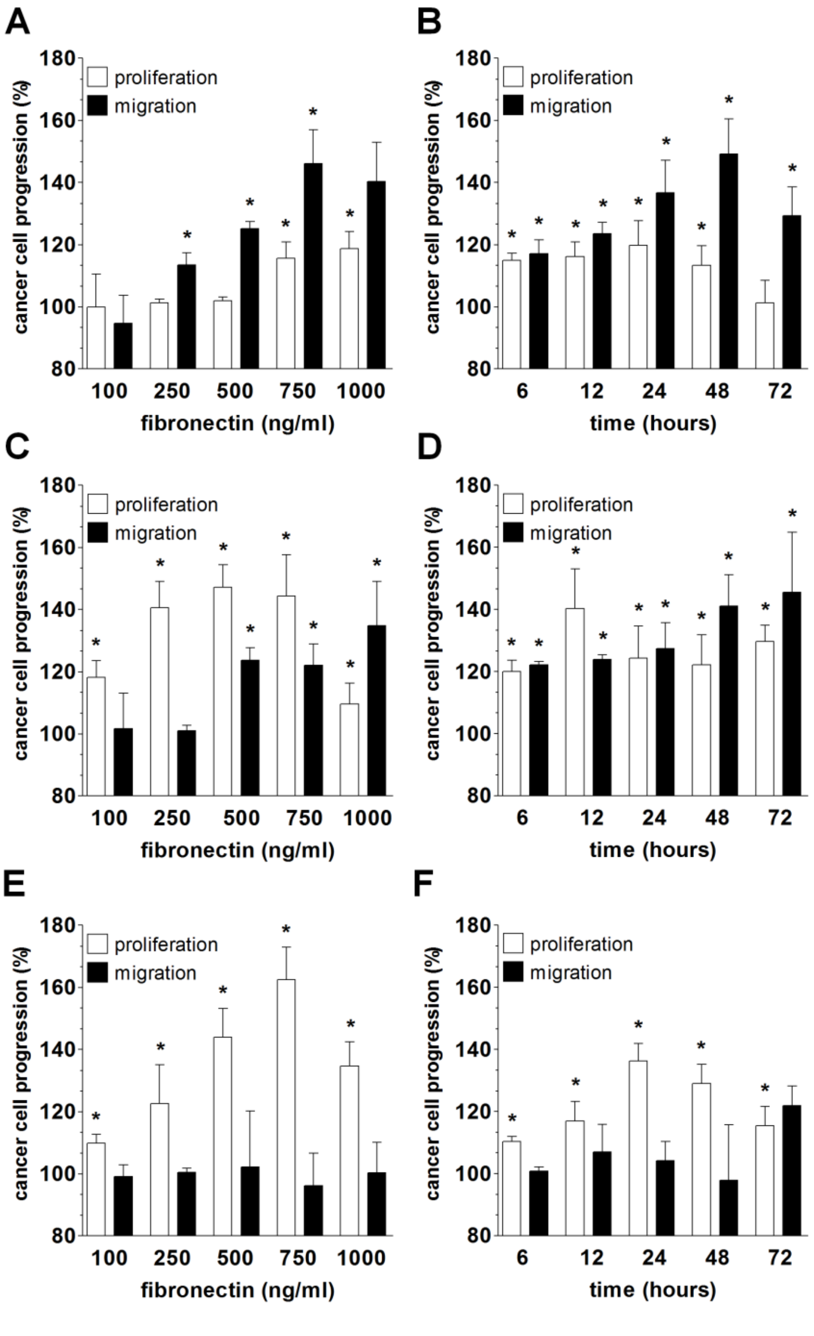

Figure S12
Effect of exogenous, recombinant form of fibronectin on proliferation and migration of ovarian cancer cells A2780 (A, B), OVCAR-3 (C, D) i SKOV-3 (D, E). Left panel (A, C, E) shows results of dose-response experiments while the right one shows results of time-course studies. The results were analysed using repeated measures analysis of variance (ANOVA) with the Newman-Keuls test as a post-hoc test. The asterisks indicate significant differences ($P<0.05$) as compared with the control, untreated cells (100%). The experiments were performed in hexaplicates. The results are expressed as mean ± SD.
